# Retraction: Medication adherence and its associated factors among type 2 diabetic patients in Ethiopian General Hospital, 2019: Institutional based cross-sectional study

**DOI:** 10.1371/journal.pgph.0001834

**Published:** 2023-04-10

**Authors:** The PLOS Global Public Health Editors

After this article [1] was published, it came to light that the authors did not have permission to use the MMAS^®^-8 scale [2], which was used in the reported study to collect medication adherence data.

This issue has not been resolved following discussions between PLOS and the authors. Therefore, the *PLOS Global Health* Editors retract this article. Considering the nature of the concerns in this case, the article contents were removed from the journal’s website at the time of retraction.

The authors did not reply or could not be reached to comment on the retraction decision.

Note: the MMAS^®^-8 scale (used in this study) was originally published in 2008 [2], but the copyright for the scale was registered by Donald E. Morisky on September 21, 2018 (U.S. Copyright Registration No. TX0008632533/2018-09-21). The MMAS^®^ trademark was also registered by Donald E. Morisky on January 29, 2019 (Reg. No. 5837374). Any reference to “MMAS” in this article [1] is for research fair use purposes.
